# *Nocardia farcinica* brain abscess in a patient with Evans syndrome: challenges in management

**DOI:** 10.1093/omcr/omag114

**Published:** 2026-07-08

**Authors:** Anjely Sebastian, Nitin Gupta, Pothumarthy Swathi Kiran, Barnini Banerjee, Muralidhar Varma, Vishwapriya Godkhindi, Tirlangi Praveen Kumar

**Affiliations:** Department of Infectious Diseases, Kasturba Medical College, Manipal Academy of Higher Education, Manipal, Karnataka 576104, India; Department of Infectious Diseases, Kasturba Medical College, Manipal Academy of Higher Education, Manipal, Karnataka 576104, India; Department of Infectious Diseases, Kasturba Medical College, Manipal Academy of Higher Education, Manipal, Karnataka 576104, India; Department of Microbiology, Kasturba Medical College, Manipal Academy of Higher Education, Manipal, Karnataka, India; Department of Infectious Diseases, Kasturba Medical College, Manipal Academy of Higher Education, Manipal, Karnataka 576104, India; Department of Pathology, Kasturba Medical College, Manipal Academy of Higher Education, Manipal, Karnataka, India; Department of Infectious Diseases, Kasturba Medical College, Manipal Academy of Higher Education, Manipal, Karnataka 576104, India

**Keywords:** nocardia, immune thrombocytopenia, brain abscess

## Abstract

Cerebral nocardiosis is a rare but serious infection, often seen in immunocompromised individuals. This report discusses a case of cerebral nocardiosis in a woman with diabetes and Evans syndrome on immunosuppressive therapy. She presented with neurological symptoms, including seizures and hemiparesis, and brain imaging revealed abscesses. Surgical excision was done and culture confirmed *Nocardia farcinica*, showing resistance to co-trimoxazole. Treatment included a combination of intravenous and oral antibiotics, tailored based on susceptibility. The report highlights the need for prophylaxis in immunosuppressed patients to prevent opportunistic infections like nocardiosis. This case underscores the importance of early diagnosis, individualized therapy, and careful management of immunosuppression to improve outcomes.

## Introduction

*Nocardia spp* are gram-positive filamentous bacterium that causes infections in immunocompromised patients. Pulmonary nocardiosis is the most common manifestation [1]. Brain abscesses occur in up to one-third of cases. Patients with symptomatic and large abscesses require drainage with needle aspiration or surgical resection and prolonged course of antimicrobial therapy based on susceptibility [1]. Here, we report the case of a middle-aged woman with *Nocardia farcinica* brain abscess managed with surgical excision and prolonged antimicrobial therapy.

## Case report

A 42-year-old woman with well-controlled Type 2 diabetes mellitus and Evans syndrome diagnosed six months earlier on immunosuppressive therapy with prednisolone (20 mg/day) and mycophenolate mofetil (2 g/day), presented with a two-week history of progressively worsening right-sided sensorimotor weakness and holocranial headache, followed by a generalized tonic–clonic seizure. Her GCS was 15/15 and she had no cranial nerve involvement or speech abnormalities. CNS examination revealed right sided hemiparesis with decreased sensation to pain and touch. She denied fever, rash, arthritis, photosensitivity, cough, or chest pain. There were no skin lesions. There was no history of tuberculosis exposure or pet contact.

An MRI of the brain revealed a 2.5 × 4 × 4 cm multiloculated ring-enhancing lesion in the left parietal lobe, with diffusion restriction and surrounding edema (Fig. 1a). Given the lesion’s size and multiloculated nature, she underwent a left parietal craniotomy with complete surgical excision of the abscess. Gram staining of the pus demonstrated Gram-positive filamentous bacteria, and aerobic culture subsequently identified *N. farcinica*. She also underwent CT chest which showed bilateral ground glass attenuation with septal thickening and mediastinal lymphadenopathy (Fig. 2).

**Figure 1:**
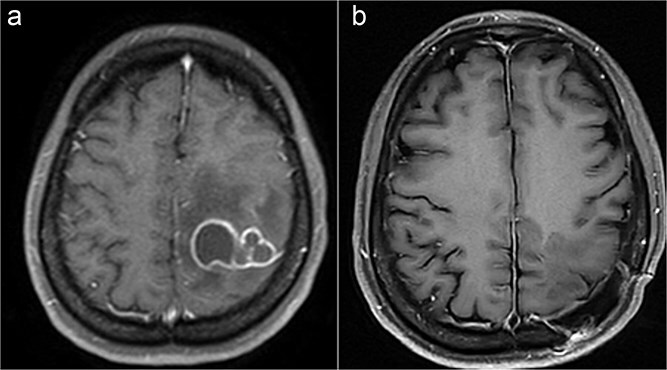
MRI brain (a) T1 sequence with contrast showing ring enhancing lesion in left parietal lobe with perilesional edema at the time of diagnosis and repeat MRI brain (b) after 6 months showing significant radiological resolution.

**Figure 2:**
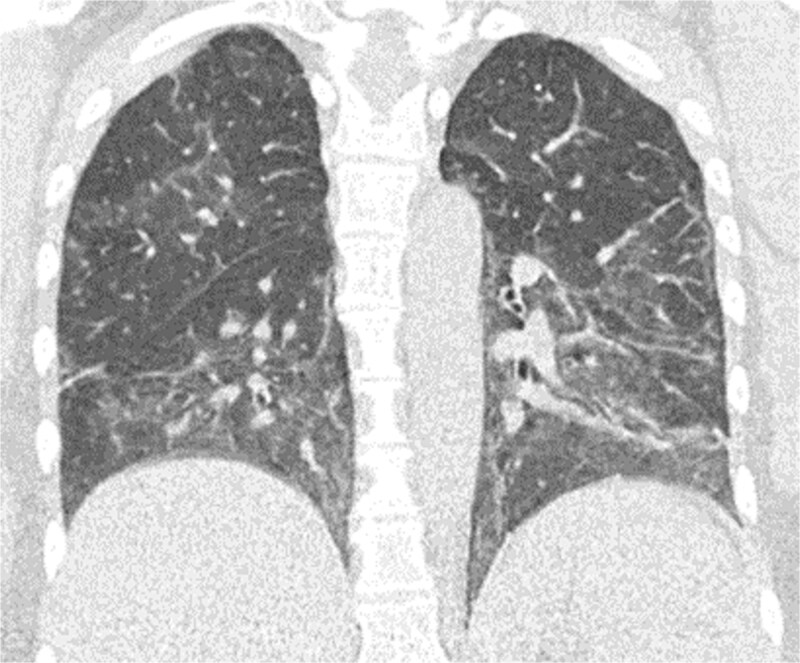
CT thorax showing bilateral ground glass attenuation suggestive of infective etiology.

**Figure 3:**
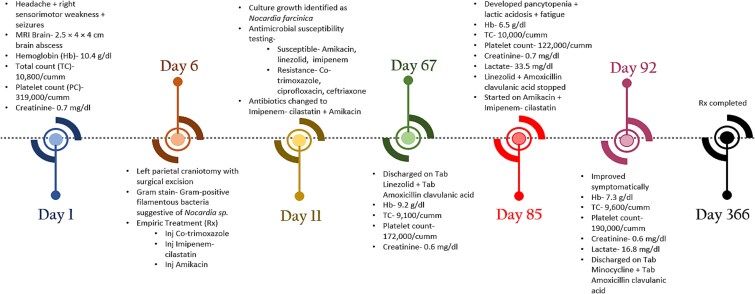
Timeline of events.

Pending susceptibility results, empiric therapy was initiated with intravenous co-trimoxazole (15 mg/kg of trimethoprim component), imipenem-cilastatin (1 g every 6 hours), and amikacin (15 mg/kg/day). Antimicrobial susceptibility testing performed using the disc diffusion method demonstrated resistance to co-trimoxazole, ciprofloxacin, and ceftriaxone, with susceptibility to amikacin, linezolid, and imipenem. As per the CLSI M24, 3rd edition guidelines, broth microdilution is the recommended method for antimicrobial susceptibility testing (AST) of *Nocardia* species. However, due to resource and manpower limitations, routine broth microdilution testing for *Nocardia* susceptibility was not performed at our center. An exception exists for sulfonamides, for which MIC results are recommended to be corroborated using the disc diffusion method. In the present case, disc diffusion testing demonstrated resistance to co-trimoxazole. Accordingly, co-trimoxazole was discontinued (except for low-dose prophylaxis against *Pneumocystis jirovecii*), and she was continued on intravenous imipenem-cilastatin and amikacin for eight weeks.

After this initial intensive phase, she was transitioned to oral linezolid (600 mg twice daily) and amoxicillin-clavulanic acid (875/125 mg twice daily). However, 18 days into linezolid therapy, she developed bicytopenia and lactic acidosis, attributed to linezolid toxicity. Linezolid was discontinued, and she briefly resumed intravenous imipenem-cilastatin and amikacin for one additional week.

Given the challenges and morbidity associated with prolonged intravenous therapy and the patient’s reluctance to continue it, oral minocycline (200 mg twice daily) was introduced alongside amoxicillin-clavulanic acid. Although initially avoided due to concerns about potential exacerbation of her autoimmune condition, minocycline was initiated with close monitoring for cytopenias, autoimmune flare, and neurological deterioration. Her clinical and radiological condition steadily improved.

At six months, follow-up MRI showed near-complete resolution of the abscess (Fig. 1b). She completed a total of 12 months of therapy without relapse. During treatment, she developed minocycline-induced tongue pigmentation, which resolved after discontinuation. Her immunosuppressive regimen was modified: mycophenolate mofetil was dose-reduced following the diagnosis of nocardiosis, and steroids were tapered gradually to 7.5 mg/day of prednisolone by six months.

## Discussion

*N. farcinica* is a catalase-positive, aerobic, Gram-positive filamentous bacterium that predominantly affects immunocompromised individuals. While pulmonary and cutaneous infections are common, central nervous system (CNS) involvement, particularly brain abscesses, occurs in up to one-third of cases. Half of such patients present with multiloculated abscesses, while others exhibit multiple cerebral abscesses or small periventricular lesions with meningeal involvement. CNS nocardiosis often presents with features of raised intracranial pressure, focal neurological deficits, or seizures. The classic triad of fever, focal deficit, and headache is observed in fewer than 10% of patients [1, 2].

Our patient, on long-term steroids and mycophenolate for Evans syndrome, developed a single multiloculated abscess in the left parietal lobe, presenting with contralateral sensorimotor deficits and seizures. Given the lesion’s size and location, surgical excision was preferred over aspiration [3]. In addition to facilitating microbiological diagnosis, surgery also relieved symptoms related to raised intracranial pressure.

Among *Nocardia* species, *N. farcinica* is notable for its neurotropism and a higher likelihood of antimicrobial resistance. Resistance to co-trimoxazole has been reported in 1–30% of *N. farcinica* isolates [4–6]. In our case, resistance to co-trimoxazole necessitated alternative therapy. Although linezolid is effective against *N. farcinica*, our patient developed significant toxicity including pancytopenia and lactic acidosis.

Due to limited oral options, minocycline was eventually chosen despite initial concerns about triggering autoimmune disease exacerbation. Though the exact pathophysiology is unknown, minocycline induced immunological adverse reaction can be classified into serum sickness, autoimmune hepatitis, vasculitis and lupus like syndromes, none of which the patient developed [7]. She tolerated the drug well under close supervision and showed clinical and radiologic improvement.

Our case has some limitations. This patient had pulmonary consolidation, suggestive of possible pulmonary nocardiosis. However, bronchoalveolar lavage was deferred because of increased intracranial pressure and the presence of a confirmed microbiological diagnosis from the brain abscess specimen.

This case highlights the complexity of treating *N. farcinica* brain abscess, especially in patients on immunosuppression. The choice of long-term oral therapy becomes particularly challenging when the organism is resistant to co-trimoxazole, and linezolid toxicity limits its use. Minocycline, though used cautiously, proved to be a viable alternative in our patient.
